# Measurement of birth outcomes in analyses of the impact of maternal influenza vaccination

**DOI:** 10.1111/irv.12673

**Published:** 2019-08-19

**Authors:** Melissa A. Rolfes, Phouvanh Vonglokham, Viengphone Khanthamaly, Bounlap Chitry, Vathsana Pholsena, Visith Chitranondh, Sara A. Mirza, Ann Moen, Joseph S. Bresee, Anonh Xeuatvongsa, Sonja J. Olsen

**Affiliations:** ^1^ Influenza Division Centers for Disease Control and Prevention Atlanta Georgia; ^2^ Ministry of Health Vientiane Lao People's Democratic Republic; ^3^ Influenza Program U.S. CDC‐Lao PDR, American Embassy Vientiane Lao People's Democratic Republic; ^4^ Mother and Child Hospital Vientiane Lao People's Democratic Republic; ^5^ Setthathirat Hospital Vientiane Lao People's Democratic Republic; ^6^ Luang Prabang Provincial Hospital Luang Prabang Lao People's Democratic Republic

**Keywords:** birth outcomes, epidemiology, influenza vaccine, maternal vaccination

## Abstract

**Background:**

The estimated association of maternal influenza vaccination and birth outcomes may be sensitive to methods used to define preterm birth or small‐for‐gestational age (SGA).

**Methods:**

In a cohort of pregnant women in Lao People's Democratic Republic, we estimated gestational age from: (a) date of last menstrual period (LMP), (b) any prenatal ultrasound, (c) first trimester ultrasound, (d) Ballard Score at delivery, and (e) an algorithm combining LMP and ultrasound. Infants were classified as SGA at birth using a Canadian, global, and equation‐based growth reference. We estimated the association of maternal influenza vaccination and birth outcomes, by influenza activity, using multivariable log‐binomial regression and Cox proportional hazards regression with vaccination as a time‐varying exposure.

**Results:**

The frequency of preterm birth in the cohort varied by method to estimate gestational age, from 5% using Ballard Score to 15% using any ultrasound. Using LMP, any ultrasound, or the algorithm, we found statistically significant reductions in preterm birth among vaccinated women during periods of high influenza activity and statistically significant increases in SGA, using a Canadian growth reference. We did not find statistically significant associations with SGA when using global or equation‐based growth references.

**Conclusions:**

The association of maternal influenza vaccination and birth outcomes was most affected by the choice of a growth reference used to define SGA at birth. The association with pre‐term birth was present and consistent across multiple statistical approaches. Future studies of birth outcomes, specifically SGA, should carefully consider the potential for bias introduced by measurement choice.

## 1 INTRODUCTION

Pregnant women and their newborns are particularly vulnerable to severe or complicated influenza virus infection. From randomized clinical trials, influenza vaccination reduces the incidence of influenza among pregnant women and their infants.^1^, ^2^, ^3^, ^4^ Some studies have further evaluated whether maternal influenza vaccination was associated with birth outcomes, including preterm birth and babies born small‐for‐gestational age (SGA). Though findings have been mixed, with some suggesting maternal vaccination reduced the risk of preterm delivery or was associated with SGA, while others have found no association.^4^, ^5^, ^6^, ^7^, ^8^, ^9^, ^10^, ^11^, ^12^ Influenza vaccination could, theoretically, prevent adverse birth outcomes by preventing potentially harmful inflammatory responses related to influenza virus infection. Though rarely seen in vaccine safety surveillance, detrimental effects of influenza vaccination, such as increased risk of miscarriage, preterm birth, or babies born small‐for‐gestational age, have also been investigated.^13^, ^14^, ^15^, ^16^ The differing study conclusions may reflect true differences, perhaps related to population, circulating strains, or vaccine match. However, we hypothesized that some of the differences are due to variability in measurement of birth outcomes, which rely on accurate estimation of gestational age and an appropriate growth reference.

Gestational age is estimated in several ways: reported date of last menstrual period (LMP), measurements of fetal size by prenatal ultrasound, or assessment of developmental features of the newborn shortly after birth. Discussions on the best methods for determining gestational age are not new, and many papers describe the differences and biases of various methods and algorithms.^17^, ^18^, ^19^, ^20^, ^21^, ^22^ Standard practice in most developed countries is to estimate gestational age based on an ultrasound examination early during pregnancy (typically before 14 weeks’ gestation). However, in resource‐limited settings where ultrasound is uncommon, observational studies may have to rely on LMP, which is prone to over‐estimation of gestation and possible misclassification of term births.^18^ Still other studies have used a combination of methods.^3^, ^23^, ^24^ Our concern was that the method used to estimate gestational age in studies of the association of influenza vaccination with birth outcomes could contribute to the variability in previous studies’ conclusions.^20^ Additional variability could come from reference birth weights used to estimate whether a baby is born small‐for‐gestational age (SGA) as several dozen growth references exist and using one reference or another can lead to very different frequencies of SGA and measured effect sizes.^25^

Furthermore, several recent discussions have suggested that uncontrolled biases (eg, healthy vaccination effect, immortal time bias) in observational studies of influenza vaccination during pregnancy may account for some or all of the observed associations with birth outcomes.^26^, ^27^, ^28^, ^29^, ^30^ These discussions encouraged re‐analysis of observational cohort data, applying statistical models that appropriately account for changing patterns of influenza circulation and the time‐dependent nature of vaccination status during pregnancy. Thus, we sought to explore how the method to estimate gestational age, different reference growth curves, and the statistical analysis approach could affect the estimated association between maternal influenza vaccination and birth outcome.

## 2 METHODS

We re‐analyzed a retrospective cohort of pregnant women in Lao People's Democratic Republic (Lao PDR) aimed at estimating the association between influenza vaccination and birth outcomes; methods were described previously.^8^ Briefly, we enrolled women into the cohort at the time of delivery at three hospitals located in Vientiane Capital and Luang Prabang provinces during April 2014‐February 2015. Health authorities conducted a national seasonal influenza vaccination campaign during 20 March‐30 June 2014. We included all women who delivered a singleton, live‐born infant and had documented vaccination status. In sensitivity analyses, we restricted the cohort to women who had a documented date of LMP, an ultrasound performed as part of her prenatal care, and whose infant had a Ballard Score performed following delivery. We collected demographic, clinical, and prenatal information from medical chart abstraction, interviews with participating women, and from an antenatal care (ANC) booklet, which documented medical visits and vaccination status.

**Figure 1:**
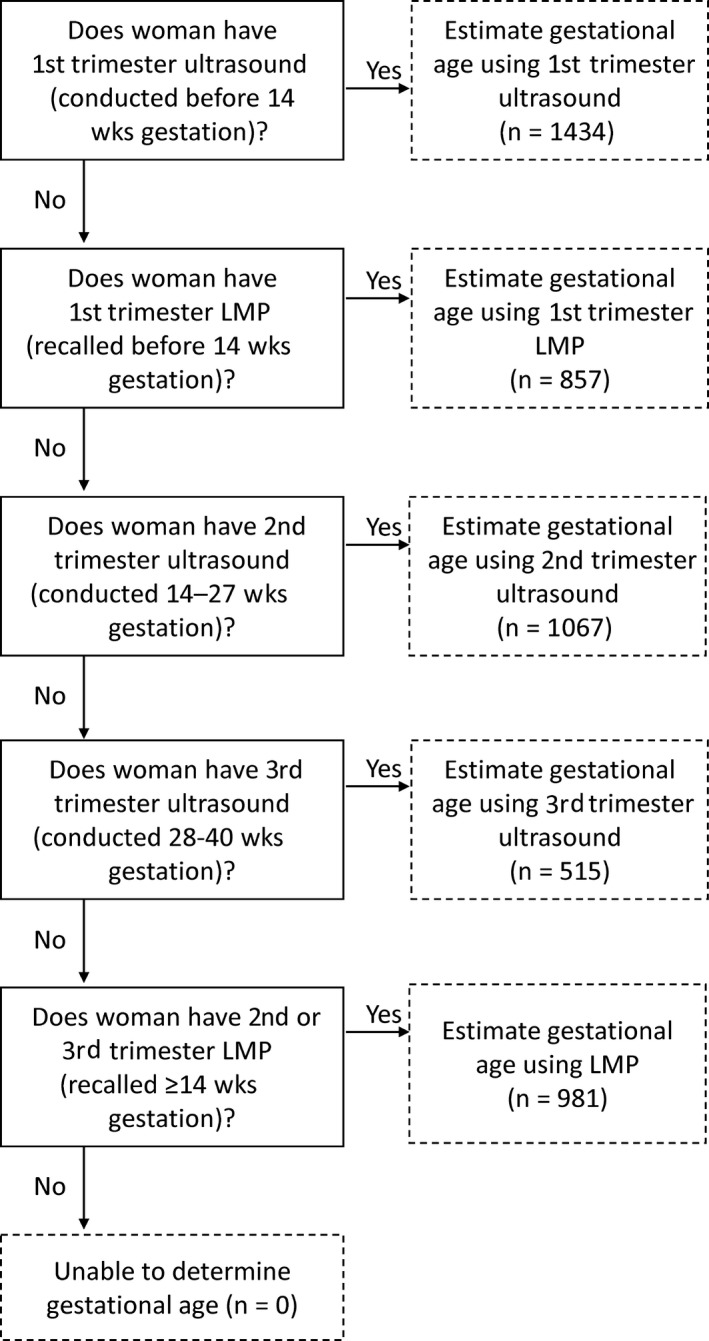
Algorithm to estimate gestational age using ultrasound or recalled last menstrual period (LMP).^23^

We estimated gestational age using five methods: (a) date of last menstrual period (LMP) as recalled by women at her first ANC visit, (b) first ultrasound conducted at any time during pregnancy, (c) first ultrasound conducted before 14 weeks of gestation, (d) Ballard Score from examination of neuromuscular and physical characteristics of the infant at birth,^31^ and (e) an algorithm that favored a first ultrasound during first trimester, followed by LMP recalled during 1st trimester, a first ultrasound during 2nd trimester, a first ultrasound during 3rd trimester, and LMP recalled during the 2nd or 3rd trimester (Figure 1).^23^ Physicians were trained on the use of Ballard Score for general practice and for the purposes of this study. We estimated the frequency of preterm delivery (delivery before 37 weeks’ gestation) among singleton, live‐born infants born after 22 weeks’ gestation.

We considered babies’ SGA when the infant's birth weight was in the lowest 10th percentile of a reference. Three reference growth charts were used: (a) a Canadian reference,^32^ (b) Intergrowth‐21st growth standard,^33^ and (c) an equation‐based method that uses the mean and standard deviation from babies born to unvaccinated mothers in the Laotian cohort.^34^

We examined the association between influenza vaccination during pregnancy and preterm birth and SGA using log‐binomial models adjusted for mother's age, parity, education, residential province, ethnic group, household income, number of antenatal care visits, number of people residing in the household, distance of the home to the hospital, and level of influenza activity at time of delivery. For the model of preterm birth, we further restricted vaccine exposure to women who were vaccinated at least 2 weeks prior to term (ie, before 35 weeks). We considered a woman unvaccinated for the analysis of preterm birth if she was vaccinated after 35 weeks’ gestation.

To examine uncontrolled bias from time‐varying exposure to influenza vaccination, we constructed adjusted Cox proportional hazards models considering vaccination as a time‐varying covariate. We included the same covariates in the Cox regression as for the log‐binomial regression. For the Cox regression analysis, we truncated observation time at 37 weeks’ gestation.

We estimated the association of maternal influenza vaccination with birth outcomes for the full cohort overall and then stratified by whether the delivery date (for log‐binomial models) or pregnancy (for Cox models) was within a period of low or high influenza activity. We used sentinel surveillance data for influenza‐like illness and influenza testing to define periods of low influenza activity (<15% of samples were influenza‐positive by reverse transcriptase polymerase chain reaction [RT‐PCR]) and high influenza activity (≥15% of samples were influenza‐positive by RT‐PCR).^35^

We conducted analyses using SAS^®^ version 9.4 (SAS Institute) and R software (version 3.1.1).^36^

## 3 RESULTS

We have published a detailed description of the cohort elsewhere.^8^ Briefly, during 2 April 2014‐27 February 2015, we enrolled 6668 women and 4854 (73%) fully met eligibility criteria, had documented vaccination records, and delivered a single live‐born infant. Based on reported LMP, women had their first antenatal care (ANC) visit at a median of 12 weeks into their pregnancy and 60% had their first ANC visit during the first trimester (Table 1). Of 4854 women, 2678 (55%) women had a documented date of LMP, an ultrasound performed during pregnancy, and an infant scored by Ballard Score (Figure S1). Using the algorithm, we estimated gestational age for all women in the cohort. Forty percent of women who had an ultrasound had one during the first trimester.

**Table 1:** Demographic and prenatal characteristics and vaccination status in a cohort of pregnant women in Lao PDR—April 2014‐February 2015

Number of women	4854
Demographic characteristics	
Median age, years (IQR)	26 (23‐30)
Median gravida (IQR)	2 (1‐3)
Median parity (IQR)	1 (0‐1)
Education level, n (%)	
None or some primary school	385/4838 (8)
Completed primary, some secondary	1723/4838 (36)
Completed secondary or higher	2730/4838 (56)
Household income >1 million Kip, n (%)	1180/4851 (24)
Prenatal characteristics	
Number of antenatal visits, median (IQR)	6 (5‐6)
≥4 antenatal visits, n (%)	3817/4568 (84)
Median estimated gestational age at first antenatal visit, LMP‐based (IQR)	12 (8‐17)
Had first ANC visit during first trimester, LMP‐based, n (%)	2040/3423 (60)
Had recalled LMP, n (%)	3707/4854 (76)
Had ultrasound, n (%)	3563/4854 (73)
First ultrasound during first trimester, n (%)	1434/3555 (40)^a^
First ultrasound during second trimester, n (%)	1423/3555 (40)
First ultrasound during third trimester, n (%)	698/3555 (20)
Had recalled LMP and ultrasound, n (%)	2913/4854 (60)
Ballard Score performed, n (%)	4368/4854 (90)
Influenza vaccination	
Vaccinated for influenza, n (%)	2142/4854 (44)
Trimester of vaccination, LMP‐based, n (%)	
First	503/1733 (29)
Second	895/1733 (52)
Third	335/1733 (19)
Trimester of vaccination, ultrasound‐based, n (%)	
First	469/1763 (27)
Second	971/1763 (55)
Third	323/1763 (18)

Abbreviations: IQR, interquartile range; LMP, last menstrual period.

a Eight women had data to indicate that an ultrasound was performed but did not have data on when the first ultrasound occurred during her pregnancy.

Women who had an ultrasound during pregnancy, compared to women who did not, were more likely to have completed secondary education (61% vs 45%; *P*‐value < .001), had ≥4 ANC visits (90% vs 61%; *P*‐value < .001), high household income (80% vs 65% with >1 million Kip; *P*‐value < .001), and received influenza vaccination (52% vs 21%; *P*‐value < .001). There were similar differences between women who could and could not recall their LMP and for women who had an early ultrasound (Table S1).

**Figure 2:**
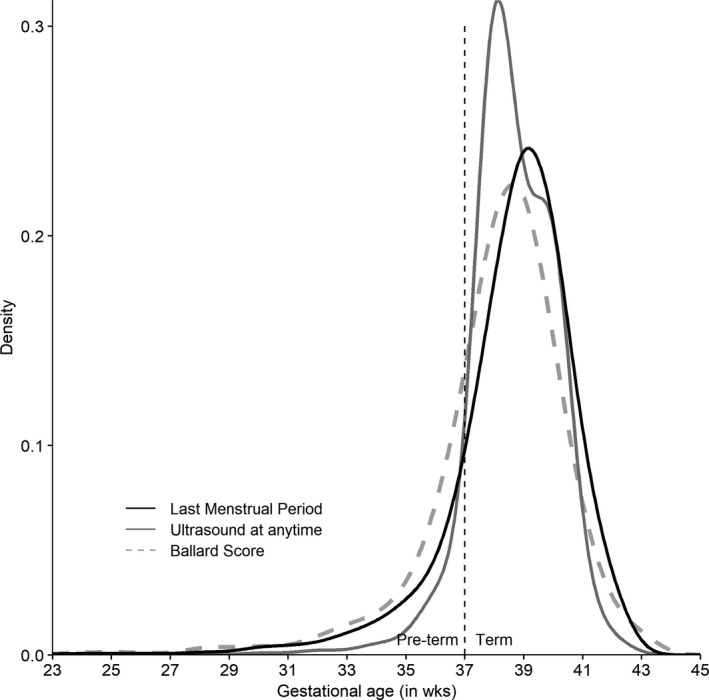
Distribution of gestational ages (GA) at birth estimated by last menstrual period, ultrasound anytime during pregnancy, or Ballard Score in children of a cohort of pregnant women in Lao PDR—April 2014‐February 2015

On average, gestational ages from ultrasound were lower than those estimated from LMP or Ballard Score (Figure 2). The frequency of preterm birth ranged from 5% to 15% and was greater when gestational age was estimated from ultrasound than from LMP, Ballard Score, or the combination of LMP and ultrasound following the algorithm (Table 2). From multivariable regression, influenza vaccination during pregnancy lowered the risk of preterm delivery when gestational age was estimated by LMP (relative risk (RR) = 0.71, 95% confidence interval (CI): 0.58‐0.88) or ultrasound at any time during pregnancy (RR = 0.69, 95% CI: 0.58‐0.83; Table 2). Using Ballard Score as the basis for gestational age resulted in a non‐significant association between influenza vaccination and preterm birth (RR = 0.89, 95% CI: 0.67‐1.17). Also, estimating gestational age from ultrasounds conducted during the first trimester resulted in a non‐significant association and a point estimate closer to the null hypothesis (RR = 0.86, 95% CI: 0.66‐1.12). Combining the LMP and ultrasound in an algorithm yielded a significant association that was closer to the null than the point estimate using LMP or ultrasound alone (RR = 0.78, 95% CI: 0.67‐0.92).

**Table 2:** Frequency of babies born preterm or small‐for‐gestational age and association with influenza vaccination during pregnancy, by method to estimate gestational age and growth reference, among pregnant women in Lao PDR—April 2014‐February 2015

Method for estimating gestational age	Live births^b^	Frequency of outcome, n (%)			Adjusted RR (95% CI)^a^		
		All	Unvaccinated	Vaccinated	Overall	High influenza activity	Low influenza activity
Preterm birth							
Last menstrual period	3691	382 (10)	247 (13)	135 (8)	0.71 (0.58‐0.88)	0.69 (0.55‐0.86)	0.89 (0.54‐1.47)
Ultrasound anytime during pregnancy	3306	487 (15)	279 (18)	208 (12)	0.69 (0.58‐0.83)	0.67 (0.56‐0.81)	1.03 (0.66‐1.61)
Ultrasound before 14 wk’ gestation	1363	195 (14)	81 (16)	114 (13)	0.86 (0.66‐1.12)	0.79 (0.59‐1.05)	—c
Ballard Score at delivery	4355	237 (5)	147 (6)	90 (4)	0.89 (0.67‐1.17)	0.87 (0.65‐1.18)	0.99 (0.48‐2.03)
Algorithm^d^	4164	585 (14)	354 (16)	231 (12)	0.78 (0.67‐0.92)	0.73 (0.61‐0.87)	1.16 (0.77‐1.74)
Small‐for‐gestational age							
Canadian growth reference							
Last menstrual period	3682	885 (24)	400 (21)	485 (27)	1.25 (1.11‐1.40)	1.22 (1.07‐1.39)	1.35 (1.03‐1.78)
Ultrasound anytime during pregnancy	3304	607 (18)	257 (16)	350 (20)	1.22 (1.05‐1.41)	1.18 (1.01‐1.39)	1.42 (0.95‐2.12)
Ultrasound before 14 wk’ gestation	1363	247 (18)	81 (16)	166 (19)	1.14 (0.89‐1.45)	1.15 (0.88‐1.49)	1.22 (0.58‐2.59)
Ballard Score at delivery	4353	894 (21)	453 (19)	441 (22)	1.15 (1.01‐1.30)	1.13 (0.99‐1.29)	1.40 (0.93‐2.13)
Algorithm^d^	4162	805 (19)	387 (18)	418 (21)	1.17 (1.03‐1.33)	1.17 (1.02‐1.35)	1.12 (0.83‐1.52)
Intergrowth‐21st growth standard							
Last menstrual period	3363	488 (15)	249 (14)	239 (15)	1.03 (0.87‐1.22)	0.99 (0.83‐1.20)	1.28 (0.84‐1.96)
Ultrasound anytime during pregnancy	3272	382 (12)	178 (11)	204 (12)	1.03 (0.84‐1.25)	1.02 (0.82‐1.26)	1.04 (0.64‐1.70)
Ultrasound before 14 wk’ gestation	1346	148 (11)	47 (10)	101 (12)	1.22 (0.88‐1.71)	1.24 (0.87‐1.79)	1.51 (0.59‐3.90)
Ballard Score at delivery	4356	416 (10)	223 (9)	193 (10)	1.01 (0.83‐1.23)	1.02 (0.83‐1.26)	1.04 (0.55‐1.97)
Algorithm^d^	4139	516 (12)	289 (13)	227 (11)	0.90 (0.76‐1.06)	0.90 (0.75‐1.09)	0.87 (0.57‐1.31)
Equation‐based growth reference							
Last menstrual period	3576	275 (8)	131 (7)	144 (8)	1.14 (0.90‐1.44)	1.04 (0.80‐1.36)	1.71 (1.00‐2.95)
Ultrasound anytime during pregnancy	3212	167 (5)	73 (5)	94 (6)	1.14 (0.84‐1.55)	1.13 (0.80‐1.58)	—c
Ultrasound before 14 wk’ gestation	1326	64 (5)	19 (4)	45 (5)	1.21 (0.72‐2.04)	1.24 (0.69‐2.22)	—c
Ballard Score at delivery	4324	226 (5)	124 (5)	102 (5)	0.98 (0.74‐1.29)	0.96 (0.71‐1.28)	1.22 (0.51‐2.94)
Algorithm^d^	4060	237 (6)	120 (6)	117 (6)	1.03 (0.80‐1.34)	1.04 (0.78‐1.38)	1.13 (0.64‐2.03)

a Adjusted relative risk (RR) and 95% confidence intervals (CI) estimated from a log‐binomial model, with robust standard errors. Model was adjusted for the mother's education, parity, age, province, and ethnicity, an indicator for ≥4 ANC visits, household income, number of household members, and distance of the mother's home to hospital. The overall model was additionally adjusted for influenza activity in the country at the time of delivery.

b Live births among women who were vaccinated at least 2 wk prior to term and women who were unvaccinated.

c The log‐binomial model failed to converge.

d The algorithm favored a first ultrasound during first trimester, followed by the last menstrual period recalled during 1st trimester, a first ultrasound during 2nd trimester, a first ultrasound during 3rd trimester, and last menstrual period recalled during the 2nd or 3rd trimester.

During periods of high influenza activity, the relative risk of preterm birth after maternal influenza vaccination was slightly further from the null hypothesis, or more protective, for all methods of estimating gestational age; and for three methods (LMP, any ultrasound, and the algorithm), the association was statistically significant. During periods of low influenza activity, none of the associations were statistically significant. When we restricted the cohort to women who had LMP, ultrasound, and Ballard Score available, the results were consistent with the findings from the full cohort (Table S2).

The frequency of infants born SGA varied considerably based on the growth reference used. Estimating gestational age using LMP, the Canadian reference resulted in the greatest frequencies of SGA (18%‐24%), followed by Intergrowth‐21st (10%‐15% SGA) and the equation‐based reference (5%‐8% SGA; Table 2). The method of estimating gestational age also had an impact on the frequency of infants born SGA, and the frequency of SGA was higher when LMP was used compared with ultrasound‐based estimates or Ballard Score. Regardless of the method used to calculate gestational age, we found that maternal influenza vaccination might increase the risk of an infant being born SGA, when the Canadian reference was used to define SGA. We also found a significant association with SGA, with the Canadian reference, when we restricted the cohort to women who delivered during a period of high influenza activity. When we used the Intergrowth‐21st standard or an equation to estimate the proportion of infants born SGA, however, there were no significant associations for any of the methods of estimating gestational age for the overall cohort nor when stratified by influenza activity.

**Figure 3:**
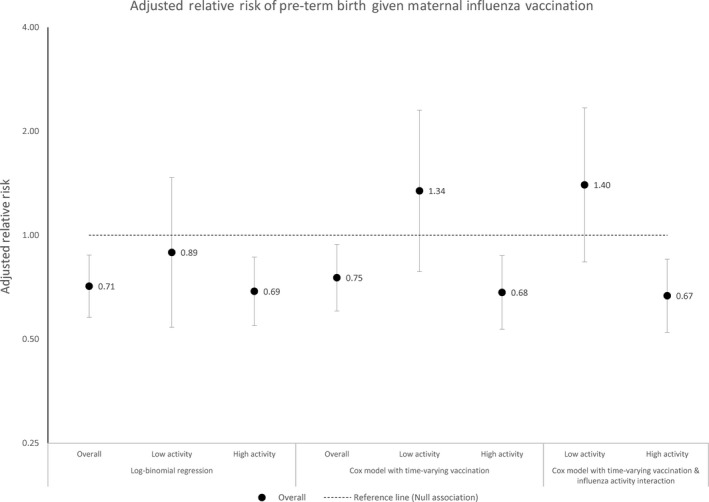
Adjusted relative risk of preterm birth after maternal influenza vaccination, by statistical model, among pregnant women in Lao PDR—April 2014‐February 2015. (Gestational age estimated using last menstrual period)

We next assessed other sources of bias in the association between maternal vaccination and birth outcome and used a Cox proportional hazards model approach as has been suggested by others.^28^ Finding little difference between using LMP or ultrasound, we chose to calculate gestational age using LMP since this was available for more women (analysis results using ultrasound and Ballard Score are shown in Figures S2 and S3). Since vaccination status for a mother can change over time, we used an adjusted Cox proportional hazards model, censoring pregnancies at 37 weeks and allowing for time‐varying vaccination status. Using this approach, our estimates of the association of maternal influenza vaccination with preterm birth were similar to the estimates from the relative risk regression for both the overall cohort and when restricting to women who delivered during periods of high influenza activity (Figure 3). We did not observe a significant association during periods of low influenza activity. We obtained very similar point estimates when we considered influenza activity as an effect modifier in the time‐varying Cox regression model.

**Figure 4:**
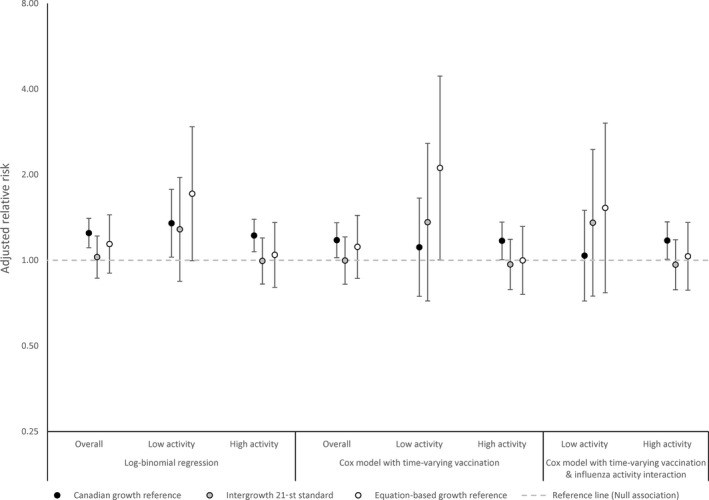
Adjusted relative risk of infants born small‐for‐gestational age after maternal influenza vaccination, by growth standard used to estimate small‐for‐gestational age and statistical model, among pregnant women in Lao PDR—April 2014‐February 2015. (Gestational age estimated using last menstrual period)

Using Cox regression for the SGA outcome also did not vary the point estimates from the relative risk regression (Figure 4). The growth reference used to define the outcome had a far greater impact on the point estimate and statistical significance.

## 4 DISCUSSION

Through re‐analysis of an existing cohort of pregnant women in Lao PDR, we sought to understand how our conclusions about the association between maternal influenza vaccination and birth outcomes varied by methods to estimate gestational age and small‐for‐gestational age (SGA), as well as the statistical models applied. We found that the frequency of preterm birth varied, yet the measured association of maternal influenza vaccination and preterm birth was unaffected, by whether the date of last menstrual period (LMP) or ultrasound at any point in pregnancy was used to estimate gestational age. When classifying infants as SGA, the choice of the growth reference affected not only the frequency of SGA in the cohort but also the magnitude and statistical significance of the association with maternal influenza vaccination.

Compared with fetal measurements by ultrasound, gestational age based on LMP tended to estimate a higher gestational age in our cohort and therefore a lower frequency of preterm birth. Others have also described this tendency and reflect that the timing of ovulation and menstruation is variable.^17^, ^18^, ^21^, ^22^, ^37^ Additionally, recall of LMP can be challenging, especially when a woman first presents to prenatal care in the later trimesters of pregnancy. However, in our data, the differences between gestational age based on LMP and that based on ultrasound did not affect the point estimates or statistical significance of the association between birth outcomes and maternal influenza vaccination.

Ultrasounds conducted early during pregnancy are the most accurate estimate of gestational age and are recommended for clinical practice.^38^, ^39^ We did not find a significant association between influenza vaccination and preterm birth when we assessed gestational age using a first trimester ultrasound. This subgroup analysis may be less influenced by measurement error; however, only 30% (1434/4854) of women in our setting had an early ultrasound, which may have limited the statistical power to detect an association between maternal influenza vaccination and birth outcomes. Also, women who had an ultrasound early in pregnancy were not representative of the full cohort, reducing the generalizability of findings and, potentially, introducing selection bias.

We found, along with others, that Ballard Score was an insensitive measure for preterm birth, as it classified half as many preterm births as LMP and ultrasound.^40^, ^41^, ^42^, ^43^ We found that associations of influenza vaccination with preterm birth and SGA were closer to the null when we used Ballard Score to determine gestational age.

Because not all women in our cohort had both a recorded LMP and an ultrasound, it was tempting to use whatever a woman had available to estimate gestational age in order to increase sample size and reduce possible selection bias. However, if vaccination status is associated with having an ultrasound or recalling LMP, as in our cohort, then the accuracy of the gestational age measurement would be associated with exposure, thereby introducing bias through classic differential misclassification. Because of this potential for bias, it is prudent to use a single measure for gestational age rather than a mixed approach for observational studies. However, we also recognize the potential for selection bias if analysis is confined to only those women who have the measured gestational age and remind the research community to be careful in designing cohort studies and use data sources with complete information.

When we looked at the association with SGA, we saw slight differences in the results by gestational age measure; however, the growth reference used had a much greater effect on our estimate of the association. For initial publication, we compared birth weights to a Canadian growth reference and found that 24% of infants in the cohort were born SGA, basing gestational age on LMP.^8^ The Canadian growth reference was chosen because of its similarity to growth references from the United States, which are commonly used. Additionally, the Canadian reference is accessible and robust; the reference has been recently updated, was population‐based, relied on early ultrasounds, and corrected for implausible values.^32^ The frequency of SGA in the Laotian cohort, using the Canadian reference, was consistent with other estimates in the region using an American reference.^44^ However, the frequency of SGA was far smaller (8%‐15%) when we use a global standard, such as Intergrowth‐21st or an equation as a reference.^33^, ^34^ When we re‐analyzed the Laotian cohort using Intergrowth‐21st or the equation‐based growth reference, we no longer see a significant association between maternal influenza vaccination and SGA. We also observed that the point estimates of the association are closer to the null, suggesting that the difference in results was not solely related to a loss of statistical power.

Others have commented on the wide variety of growth references available and how variable the frequency of SGA infants can be.^25^, ^45^, ^46^ Global standards, such as the WHO equation or Intergrowth 21st, which reflect the range of diversity in genetics, prenatal exposures, and maternal health seen worldwide, may be more appropriate for studies in countries that do not have validated growth charts and for studies that will eventually be compared across countries, as has been the case for the association of maternal influenza vaccination with birth outcomes.

We also explored whether the choice of analytic approach could be another source of variability, as has been suggested by others.^28^, ^29^, ^30^ We initially analyzed the cohort using a log‐binomial model to estimate a relative risk of each outcome by maternal vaccination; therefore, we were not surprised that our initial point estimates for the association varied little from those obtained from the Cox proportional hazards model using time‐varying vaccination status. Certainly, estimates of the association could be biased if vaccination were treated as time‐independent exposures in a Cox regression approach.

In summary, through re‐analysis of a cohort of pregnant women, we found that the association of influenza vaccination during pregnancy with preterm birth and SGA was not sensitive to whether LMP or ultrasound was used to estimate gestational age, as long as the same method was used for all members of the cohort. The association with SGA was sensitive to the growth reference, and employing a global growth standard in future analyses would allow for cross‐country comparisons and may be more appropriate for countries that do not have an existing local growth reference. Furthermore, alternative analytic strategies did not change the associations with either pre‐term birth or SGA. Future analyses should also use sound statistical models to investigate the association of birth outcomes with maternal influenza vaccination. Although discussion of appropriate measures of gestational age and birth outcome is not new, during this formative time in the discussion of maternal influenza vaccination and its possible beneficial impact on birth outcomes it is important to maintain a critical eye on the methodological issues and potential biases in conducting and analyzing observational cohorts.
