# Supplementary material for: Measurement of birth outcomes in analyses of the impact of maternal influenza vaccination
Source: Influenza Other Respir Viruses. 2019 Aug 19;13(6):547–55. doi: 10.1111/irv.12673 (PMC6800304; doi:10.1111/irv.12673)
Supplement: Supplementary file 1 [file IRV-13-547-s001.docx]

**Measurement of Birth Outcomes in Analyses of the Impact of Maternal Influenza Vaccination**

**Supplemental Figure 1:** Methods of estimating gestational age used in a cohort of pregnant women in Lao People’s Democratic Republic —April, 2014–February, 2015.


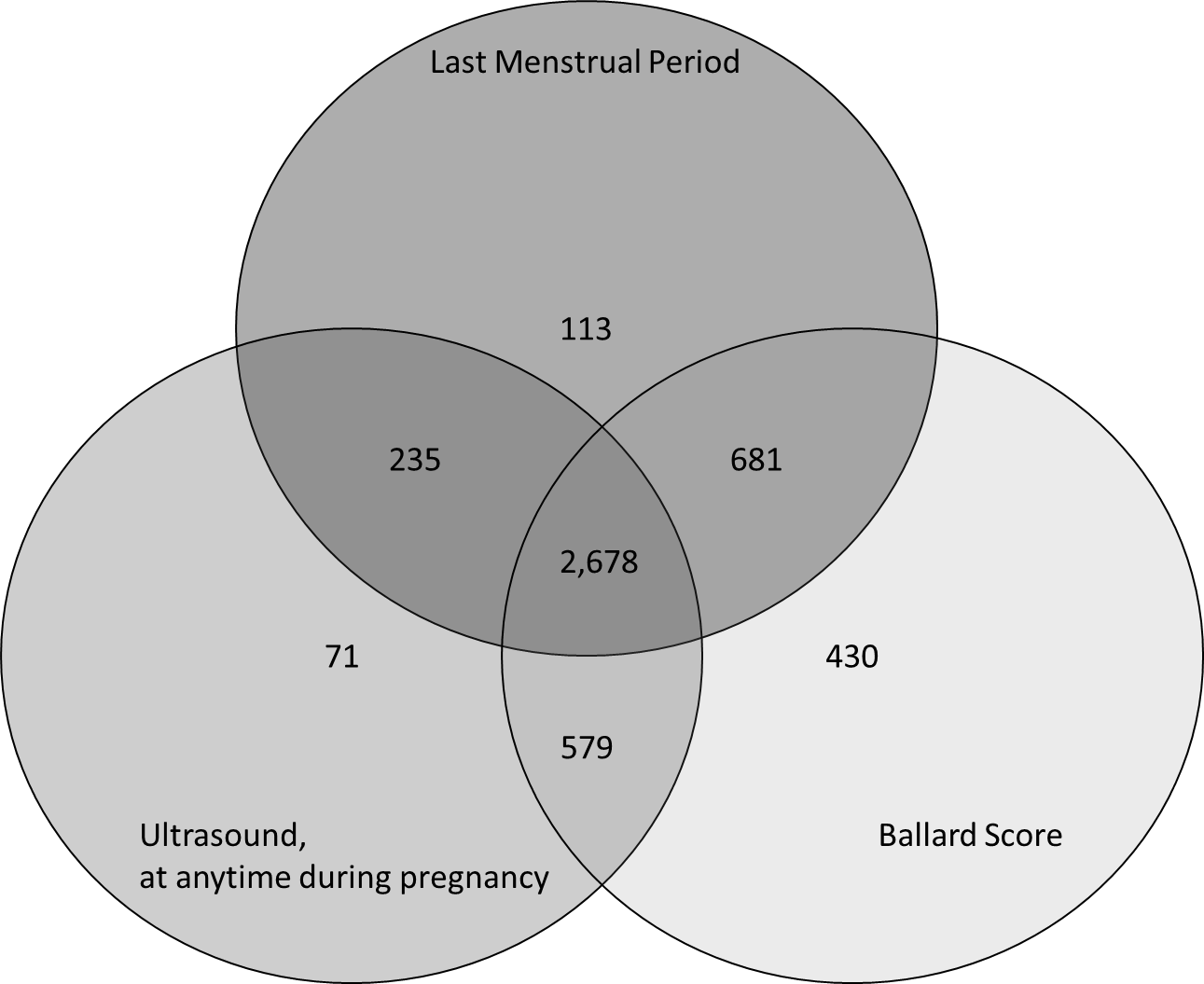


**Supplemental Figure 2:** Adjusted relative risk of preterm birth with maternal influenza vaccination, using ultrasound to estimate gestational age in a cohort of pregnant women in Lao People’s Democratic Republic —April, 2014–February, 2015.
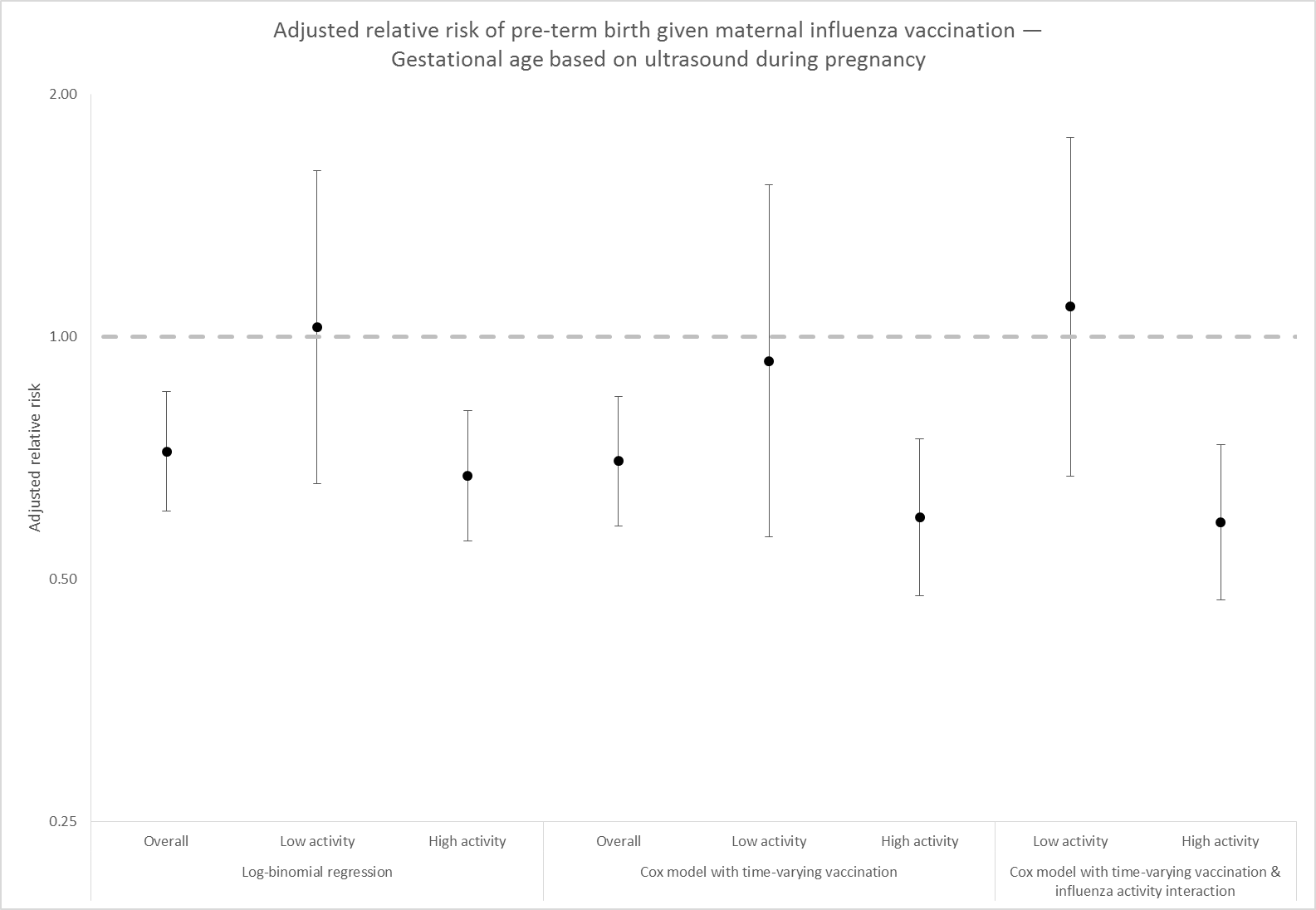


**Supplemental Figure 3:** Adjusted relative risk of preterm birth with maternal influenza vaccination, using Ballard Score at birth to estimate gestational age of the infant born to pregnant women in Lao People’s Democratic Republic —April, 2014–February, 2015.
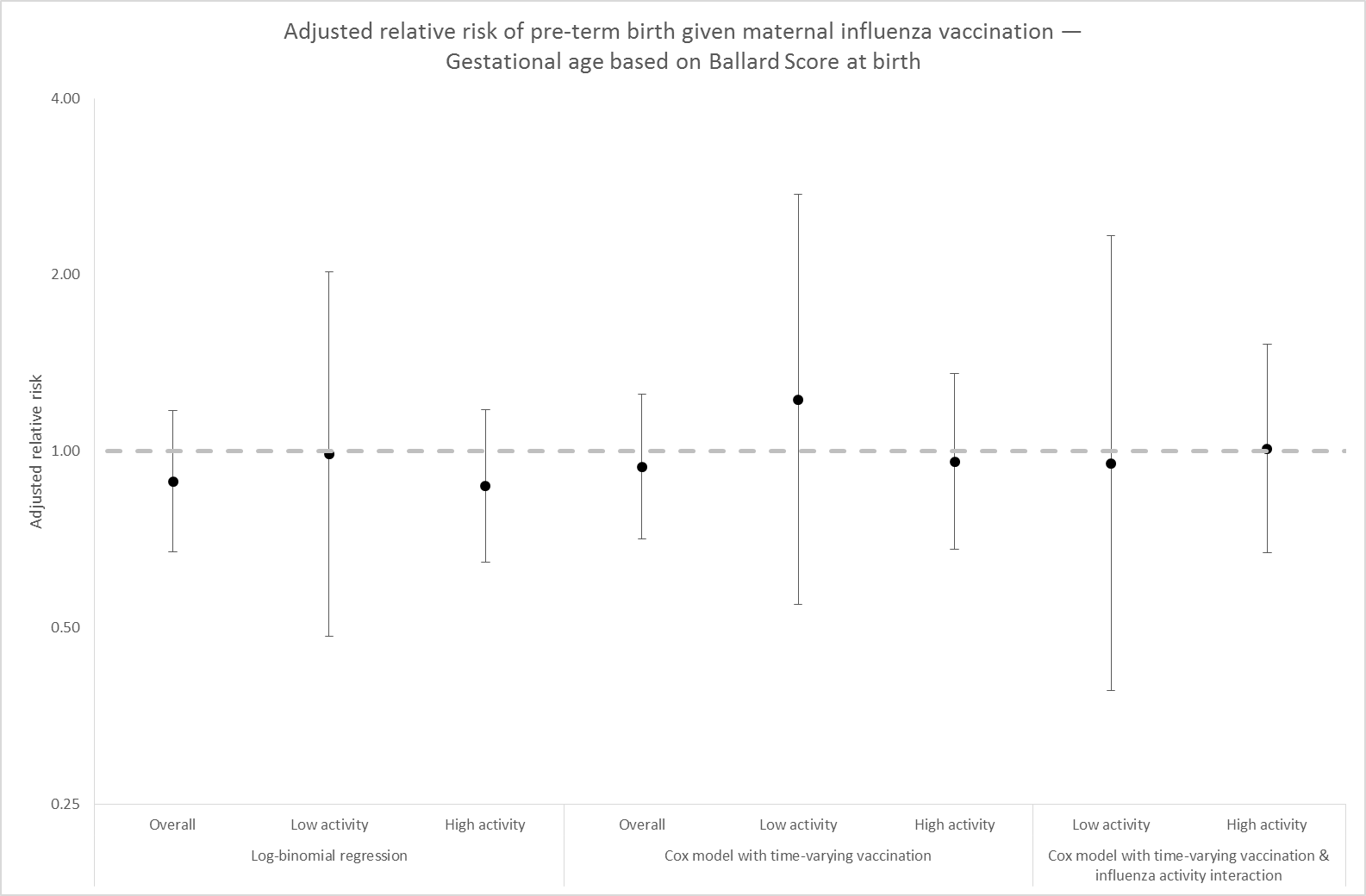


**Supplemental Table 1:** Demographic and prenatal characteristics of a cohort of pregnant women in Lao People’s Democratic Republic, by measures of gestational age used in the cohort —April, 2014–February, 2015.

|  | **All women with live births** | **All women with live births who had an ultrasound at any time during pregnancy** | **All women with live births who had an ultrasound before 14 weeks gestation** | **All women with live births who recalled LMP** | **All women with live births who recalled LMP, had an ultrasound at any time during pregnancy, and had Ballard Score** |
| --- | --- | --- | --- | --- | --- |
| Number of women | 4,854 | 3,563 | 1,434 | 3,707 | 2,678 |
| ***Demographic characteristics*** |  |  |  |  |  |
| Median age, years (IQR) | 26 (23–30) | 26 (23–30) | 27 (24–30) | 26 (23–30) | 27 (23–30) |
| Median gravida (IQR) | 2 (1–3) | 2 (1–3) | 2 (1–3) | 2 (1–3) | 2 (1–3) |
| Median parity (IQR) | 1 (0–1) | 1 (0–1) | 0 (0–1) | 1 (0–1) | 1 (0–1) |
| Education level, n/N (%) |  |  |  |  |  |
| None or some primary school | 385/4,838 (8) | 193/3,555 (5) | 38/1,431 (3) | 165/3,701 (4) | 89/2,673 (3) |
| Completed primary, some secondary | 1,723/4,838 (36) | 1,203/3,555 (34) | 407/1,431 (28) | 1,239/3,701 (33) | 861/2,673 (32) |
| Completed secondary or higher | 2,730/4,838 (56) | 2,159/3,555 (61) | 986/1,431 (69) | 2,297/3,701 (62) | 1,723/2,673 (64) |
| Household income >1M Kip, n/N (%) | 1,180/4,851 (24) | 728/3,560 (20) | 232/1,434 (16) | 790/3,705 (21) | 485/2,676 (18) |
|  |  |  |  |  |  |
| ***Prenatal characteristics*** |  |  |  |  |  |
| Number of ANC visits, median (IQR) | 6 (5–6) | 6 (5–6) | 6 (6–6) | 6 (5–6) | 6 (6–6) |
| ≥4 ANC visits, n/N (%) | 3,817/4,568 (84) | 3,096/3,398 (91) | 1,355/1,388 (98) | 3,194/3,516 (91) | 2,498/2,672 (93) |
| Median estimated GA at first ANC visit,  LMP-based (IQR) | 12 (8–17) | 12 (8–16) | 9 (7–11) | 12 (8–17) | 12 (8–16) |
| Had first ANC visit during first trimester, LMP-based, n/N (%) | 2,040/3,423 (60) | 1,708/2,749 (62) | 1,107/1,192 (93) | 2,028/3,402 (60) | 1,636/2,615 (63) |
| Had recalled LMP, n/N (%) | 3,707/4,854 (76) | 2,913/3,563 (82) | 1,252/1,434 (87) | -- | -- |
| Had ultrasound, n/N (%) | 3,563/4,854 (73) | -- | -- | 2,913/3,707 (79) | -- |
| Trimester of first ultrasound, n/N (%) |  |  |  |  |  |
| First | 1,434/3,555 (40) | 1,434/3,563 (40) | 1,434/1,434 (100) | 1,252/3,707 (43) | 1,172/ (44) |
| Second | 1,423/3,555 (40) | 1,423/3,563 (40) | -- | 1,148/3,707 (39) | 1,065/ (40) |
| Third | 698/3,555 (20) | 698/3,563 (20) | -- | 507/3,707 (17) | 437/ (16) |
| Had recalled LMP and had ultrasound, n/N (%) | 2,913/4,854 (60) | 2,913/3,563 (82) | 1,252/1,434 (87) | 2,913/3,563 (82) | -- |
| Infant had Ballard Score, n/N (%) | 4,368/4,854 (90) | 3,257/3,563 (91) | 1,336/1,434 (93) | 3,257/3,563 (91) | -- |
|  |  |  |  |  |  |
| ***Influenza vaccination*** |  |  |  |  |  |
| Vaccinated for influenza, n/N (%) | 2,142/4,854 (44) | 1,866/3,563 (52) | 916/1,434 (64) | 1,866/3,563 (52) | 1,508/2,678 (56) |
| Trimester of vaccination, LMP-based, n/N (%) | |  |  |  |  |
| First | 503/1,733 (29) | 417/1,586 (26) | 295/809 (36) | 417/1,586 (26) | 412/1,494 (28) |
| Second | 895/1,733 (52) | 840/1,586 (53) | 385/809 (48) | 840/1,586 (53) | 801/1,494 (54) |
| Third | 335/1,733 (19) | 329/1,586 (21) | 129/809 (16) | 329/1,586 (21) | 281/1,494 (19) |
| Trimester of vaccination, ultrasound-based, n/N (%) | |  |  |  |  |
| First | 469/1,763 (27) | 467/1,757 (27) | 327/873 (37) | 467/1,757 (27) | 395/1,415 (28) |
| Second | 971/1,763 (55) | 971/1,757 (55) | 429/873 (49) | 971/1,757 (55) | 790/1,415 (56) |
| Third | 323/1,763 (18) | 319/1,757 (18) | 117/873 (13) | 319/1,757 (18) | 230/1,415 (16) |

**Supplemental Table 2:** Association with influenza vaccination during pregnancy and risk of preterm birth or small-for-gestational age among 2,678 pregnant women with complete data on gestational age as estimated by date of last menstrual period, ultrasound at any time during pregnancy, and Ballard Score of her infant at birth — Lao PDR, April, 2014–February, 2015

| **Method for estimating gestational age** | **Live births** | *All* | | *Unvaccinated* | *Vaccinated* | |  | | | *Overall* | | | *High influenza activity* | | *Low influenza activity* | |
| --- | --- | --- | --- | --- | --- | --- | --- | --- | --- | --- | --- | --- | --- | --- | --- | --- |
|  |  | **Preterm birth, n (%)** | | | | | |  | | | **Adjusted RR (95% CI) ^^^** | | | | | |
| Last menstrual period | 2,645 | 235 (9) | | 135 (12) | 100 (7) | |  | | | 0.73 (0.57-0.95) | | | 0.71 (0.54-0.93) | | 1.07 (0.41-2.82) | |
| Ultrasound anytime during pregnancy | 2,498 | 354 (14) | | 203 (19) | 151 (11) | |  | | | 0.65 (0.53-0.79) | | | 0.61 (0.49-0.75) | | 1.33 (0.60-2.92) | |
| Ballard Score at delivery | 2,667 | 118 (4) | | 60 (5) | 58 (4) | |  | | | 0.88 (0.61-1.27) | | | 0.98 (0.66-1.45) | | 0.46 (0.18-1.18) | |
| Algorithm ^†^ | 2,554 | 339 (13) | | 192 (17) | 147 (10) | |  | | | 0.67 (0.55-0.83) | | | 0.64 (0.52-0.80) | | 1.18 (0.55-2.53) | |
|  |  |  | |  |  | |  | | |  | | |  | |  | |
|  |  | **Infant born small-for-gestational age, n (%)** | | | | | |  | | |  | | | | | |
| **Canadian growth standard** |  |  | |  |  | |  | | |  | | |  | |  | |
| Last menstrual period | 2,638 | 666 (25) | | 254 (22) | 412 (28) | |  | | | 1.20 (1.04-1.38) | | | 1.20 (1.04-1.39) | | 1.10 (0.71-1.69) | |
| Ultrasound anytime during pregnancy | 2,496 | 466 (19) | | 174 (16) | 292 (21) | |  | | | 1.29 (1.08-1.53) | | | 1.26 (1.05-1.51) | | 2.03 (0.90-4.54) | |
| Ballard Score at delivery | 2,665 | 568 (21) | | 223 (19) | 345 (23) | |  | | | 1.19 (1.02-1.38) | | | 1.14 (0.97-1.35) | | -- * | |
| Algorithm ^†^ | 2,552 | 489 (19) | | 184 (16) | 305 (21) | |  | | | 1.27 (1.07-1.51) | | | 1.25 (1.05-1.49) | | 1.72 (0.86-3.41) | |
| **Intergrowth 21^st^ growth standard** | |  |  | |  |  | | |  | | |  | |  | |  |
| Last menstrual period | 2,440 | 353 (14) | | 157 (15) | 196 (14) | |  | | | 0.94 (0.77-1.16) | | | 0.97 (0.78-1.20) | | 0.75 (0.42-1.33) | |
| Ultrasound anytime during pregnancy | 2,474 | 287 (12) | | 121 (11) | 166 (12) | |  | | | 1.07 (0.85-1.35) | | | 1.04 (0.82-1.32) | | 1.88 (0.77-4.56) | |
| Ballard Score at delivery | 2,669 | 255 (10) | | 107 (9) | 148 (10) | |  | | | 1.03 (0.81-1.32) | | | 1.01 (0.78-1.31) | | 1.60 (0.63-4.08) | |
| Algorithm ^†^ | 2,537 | 304 (12) | | 143 (13) | 161 (11) | |  | | | 0.87 (0.70-1.08) | | | 0.89 (0.71-1.13) | | 0.69 (0.35-1.36) | |
| **Equation-based growth standard** | |  |  | |  |  | | |  | | |  | |  | |  |
| Last menstrual period | 2,561 | 201 (8) | | 79 (7) | 122 (9) | |  | | | 1.13 (0.85-1.50) | | | 1.04 (0.78-1.40) | | 3.97 (0.97-16.16) | |
| Ultrasound anytime during pregnancy | 2,436 | 121 (5) | | 49 (5) | 72 (5) | |  | | | 1.15 (0.79-1.66) | | | 1.16 (0.79-1.69) | | -- * | |
| Ballard Score at delivery | 2,652 | 146 (6) | | 62 (5) | 84 (6) | |  | | | 1.00 (0.72-1.40) | | | 0.97 (0.68-1.36) | | 1.98 (0.45-8.70) | |
| Algorithm ^†^ | 2,504 | 135 (5) | | 54 (5) | 81 (6) | |  | | | 1.16 (0.82-1.64) | | | 1.13 (0.79-1.63) | | 2.43 (0.56-10.65) | |

^^^ Adjusted relative risk (RR) and 95% confidence intervals (CI) estimated from a log-binomial model, with robust standard errors. Model was adjusted for the mother’s education, parity, age, province, and ethnicity, an indicator for ≥4 ANC visits, household income, number of household members, and distance of the mother’s home- to hospital. The overall model was additionally adjusted for influenza activity in the country at the time of delivery.

* The log-binomial model failed to converge.

^†^ The algorithm favored a first ultrasound during first trimester, followed by the last menstrual period recalled during 1^st^ trimester, a first ultrasound during 2^nd^ trimester, a first ultrasound during 3^rd^ trimester, and last menstrual period recalled during the 2^nd^ or 3^rd^ trimester.
